# Physical activity and depressive symptoms among Chinese university students: Grit as a mediator

**DOI:** 10.1371/journal.pone.0350277

**Published:** 2026-05-28

**Authors:** Yizhou Chen, Jie Zhang

**Affiliations:** 1 Department of Physical Education, Quzhou University, Quzhou, Zhejiang, China; 2 College of Physical Education, Anhui Normal University, Wuhu, Anhui, China; University of Mpumalanga, SOUTH AFRICA

## Abstract

**Background:**

While physical activity is widely recognized for its protective role against depressive symptoms, the specific psychological mechanisms that explain this relationship, particularly among Chinese university students, require further exploration. The present study seeks to clarify whether grit as a hypothesized mediator explaining how physical activity is linked to depressive symptoms severity within this population.

**Methods:**

In this cross-sectional investigation, 3,140 Chinese university students were recruited to complete an online survey. Assessments included demographic variables, physical activity levels, grit, and depressive symptoms, each measured with validated instruments. The hypothesized mediation pathway—wherein grit serves as the mediating variable—was tested via the PROCESS macro for SPSS, with bias-corrected bootstrapping (5,000 resamples) used to assess the indirect effect.

**Results:**

Analyses demonstrated a significant negative association between physical activity and depressive symptoms. Crucially, grit was found to completely mediate this association. The indirect pathway through grit was statistically significant (*ab* = −0.013, 95% CI [−0.016, −0.010]), whereas the direct effect of physical activity was non-significant (*c’* = −0.002, *p* > 0.05, 95% CI [−0.011, 0.007]).

**Conclusions:**

These findings indicate that the beneficial effect of physical activity on depressive symptoms is primarily channeled through grit. Intervention strategies aimed at improving mental well-being in this population may be more effective if they combine physical activity promotion with grit-fostering components.

## Introduction

Depression represents a prevalent mental disorder characterized by enduring emotional and cognitive disturbances. Key diagnostic features include a persistent state of low mood or markedly diminished interest in daily activities. These core symptoms must be present for most of the day, over a minimum two-week period, and are often accompanied by disturbed sleep, appetite changes, low self-worth, suicidal thoughts, hopelessness, fatigue, and poor concentration [1,2]. Depression not only severely disrupts patients’ daily lives, work, and studies, but also places a significant burden on their families and society [3–7]. While individuals across various age brackets can experience depressive symptoms [8], college students are at a notably elevated risk [9]. Recent data points to an escalating crisis, with a growing number of university students reporting significant levels of depressive symptoms. A longitudinal study tracking 1,795 U.S. student pharmacists revealed a significant increase in depressive symptoms, with rates climbing from 57.8% prepandemic to 71.4% in 2021 [10]. Another longitudinal study on U.S. college students also indicates that their depressive symptom levels are more severe than in previous years [11]. In the Chinese context, depression has become increasingly prevalent among university students, with reported rates rising from 21.9% during 2011–2015 [12] to exceeding 30% by 2024 [13,14]. A systematic review of global data documented a marked increase in depressive symptoms in this population following the onset of the COVID-19 pandemic [15]. Depressive symptoms entail considerable risks, such as poor academic performance, for university students [16,17]. This is because it triggers emotional exhaustion and professional burnout, while also reducing students’ academic self-efficacy [5]. Depression adversely affects the social relationships of university students [18], often manifested by a heightened sense of rejection during interactions with peers [19]. More critically, depressive symptoms may lead to suicidal behaviors among university students, posing a significant threat to their life safety [20,21]. Depressive symptoms exact a severe toll on university students, exploring protective mechanisms has become a pressing priority.

A growing body of evidence underscores the protective role of physical activity in both preventing and alleviating depressive symptoms [22–24]. Concurrently, grit has gained scholarly attention as a psychologically relevant trait, with distinct cultural manifestations in Chinese societies rooted in Confucian values of perseverance, diligence, and long-term goal striving [25,26]. Cross-cultural research indicates that Chinese conceptualizations of grit are more closely tied to collectivist obligations and educational resilience compared to Western individualistic frameworks [26,27]. Empirical work by Dunston et al. [28] linked higher volumes of high-intensity physical activity among college students to elevated grit levels, a finding corroborated in a longitudinal study of 2,142 Korean adolescents, which established a positive effect of physical exercise on grit development [29]. Among Chinese samples, regular physical activity has similarly been shown to foster grit’s core components—perseverance of effort and consistency of interest—aligning with cultural virtues of resilience [30,31]. Furthermore, grit has been consistently associated with improved psychological well-being [32], with studies suggesting it strengthens stress resilience and reduces vulnerability to psychological distress and depression [33,34]. Consistent with accumulating evidence that physical activity fosters grit and that grit in turn attenuates depressive symptoms, a plausible mediating pathway is hypothesized. It is proposed that grit functions as a psychological mechanism through which physical activity exerts its beneficial effects, operating both directly and indirectly on depressive symptomatology in the student population.

Nevertheless, the interrelationship among physical activity, grit, and depressive symptoms in the university student population remains insufficiently explored. A growing body of research has verified the connection between physical activity and depressive symptoms [22,24,35], while paying limited attention to grit as a potential mediator. Although Toczko et al. [36] investigated the effects of both physical activity and grit on depressive symptoms, their findings indicated no significant link between grit and depression in the initial phase of the COVID-19 pandemic. The authors proposed that grit’s influence might become more pronounced as the pandemic continued. Moreover, empirical research concentrating specifically on Chinese university students is relatively scarce. To date, only a few studies have examined the effect of physical exercise on grit in this demographic [31,37], and similarly, a limited number have addressed the relationship between grit and depressive symptoms [34,38,39]. This scarcity constrains a nuanced understanding of how Chinese cultural and social contexts may modulate the dynamic between these variables. To address these identified gaps, this study is designed to assess the effect of physical activity on depressive symptoms in Chinese university students and to evaluate the potential mediating function of grit. Through a targeted survey, this research aims to clarify the psychological pathways linking these constructs. The outcomes are anticipated to establish an empirical foundation and inform the design of tailored mental health strategies, ultimately supporting enhanced psychological well-being in this student population.

### Theoretical background and hypotheses

#### Theoretical framework.

This research is grounded in resilience theory, which conceptualizes resilience as the capacity to withstand, recover from, and achieve positive adaptation when confronted with significant adversity [40,41]. As a central framework in positive psychology, this theory focuses on individuals’ dynamic ability to adapt and maintain well-being despite challenges such as academic pressure, emotional difficulties, or depressive symptoms [40,41]. Rather than treating resilience as a static trait, modern theoretical developments—exemplified by Masten’s perspective of “ordinary magic”—frame it as a developmental process emerging from the interplay between personal assets, environmental supports, and adaptive behaviors [40]. This process-oriented view aligns closely with the core research questions of the present study, which explores how physical activity (an adaptive behavioral factor) influences depressive symptoms (a key indicator of adversity-related distress) among Chinese university students, with grit (a stable individual protective factor) acting as a mediator. Within the resilience theory framework, physical activity operates as an activator of external resources: regular participation in activities such as aerobic exercise or team sports contributes not only to physiological health [42,43] but also cultivates internal protective factors, among which grit is particularly salient [41]. Grit, characterized by sustained perseverance and dedication to long-term objectives [44], aligns with resilience theory’s emphasis on “sustained adaptive effort” as a core component of resilience. Within this framework, grit serves as a psychological bridge: physical activity cultivates grit by requiring repeated effort, goal-setting, and overcoming challenges (e.g., maintaining a workout routine despite academic pressure). Subsequently, grit mitigates depressive symptoms by strengthening stress management capacity, sustaining motivation through emotional difficulties, and reinforcing a sense of purposeful direction—core processes through which resilience counteracts the impact of adversity [41,45]. Notably, resilience theory’s emphasis on the contextual nature of protective factors [41] justifies focusing on Chinese university students’ specific environment. For this population, who often face unique stressors (e.g., academic stress, career uncertainty, and interpersonal stress) [46–48], physical activity and grit may operate as particularly salient resilience resources: physical activity provides an accessible, low-cost avenue to build psychological resilience, while grit addresses the chronic nature of stressors (e.g., prolonged academic demands) by sustaining adaptive coping over time [40]. This study situates the “physical activity → grit → depressive symptoms” mediation model within resilience theory, thereby advancing the understanding of how actionable factors (e.g., physical activity) can be strategically harnessed to strengthen psychological resilience and alleviate depressive symptoms.

#### Physical activity and depressive symptoms.

The World Health Organization [49] defines physical activity as any bodily movement produced by skeletal muscles that requires energy expenditure. As a deeply integrated component of daily life with substantial functional importance, physical activity has garnered widespread research attention for its impacts on mental health. A large body of empirical evidence confirms its significant benefits in reducing negative affect and enhancing psychological well-being [50–52]. Substantial empirical work confirms an inverse relationship between physical activity and depressive symptomatology [53–55]. A meta-analysis of 17 studies concluded that engagement in physical activity, irrespective of its specific form, intensity, or duration, is associated with significant reductions in depressive symptoms [35]. This protective effect extends across demographic groups; for example, a meta-analysis of 24 randomized controlled trials (N = 1,128) found that exergame-based physical activity significantly alleviated depressive symptoms in older adults [56]. The long-term benefit is further corroborated by a 20-year longitudinal study of over 12,000 adults aged 50 and above, which identified a persistent inverse association between moderate physical activity and depressive symptoms [57]. Grounded in this body of evidence, the present study posits its first hypothesis (H1): Physical activity is negatively associated with depressive symptoms, wherein greater engagement in physical activity corresponds to lower levels of depressive symptom severity.

### Girt as a mediator

While the inverse correlation between physical activity and depressive symptoms is well-documented, the psychological pathways that account for this relationship require further clarification. The current research proposes grit as a pivotal explanatory mechanism underlying this association in the university student population. Defined by Duckworth and her team [44] as the combination of perseverance and sustained interest toward long-term objectives, grit reflects an individual’s ability to consistently invest effort and maintain goal-directed motivation despite difficulties, failures, or prolonged periods of limited progress [58,59]. Notably, physical activity serves as a practical context where such perseverance and passion are often tested—when engaging in physical activity, university students inevitably face various difficulties and challenges, such as constraints imposed by weather conditions, sports-related injuries, heavy academic pressure, insufficient time and energy, and low motivation for exercise [60]. Only an individual with grit is able to persist in physical activity consistently when facing these difficulties [61,62]. Through sustained efforts to overcome adversity, university students cultivate a resilient sense of efficacy, thereby forging increased psychological strength and adaptive capacities [63]—attributes that align with the core of grit. This process of shaping grit through persistent physical activity, in turn, connects to better mental health outcomes. A well-documented association exists between grit and favorable mental health outcomes. Among university students, higher grit not only predicts greater resilience to daily stressors—manifesting as reduced burnout [64] and sustained positive affect [65]—but also confers protection against clinical symptoms. This inverse relationship with psychopathology is further confirmed by its significant negative correlation with depressive symptoms [66]. Synthesizing this theoretical and empirical foundation, the present study advances its second hypothesis (H2): Grit serves as a mediating variable in the association between physical activity and depressive symptoms, wherein physical activity contributes to symptom reduction through the enhancement of grit.

**Fig 1:**
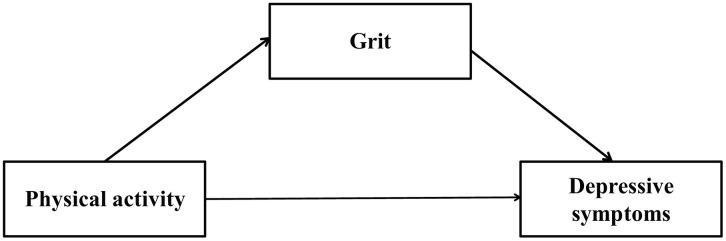
Proposed mediational model linking physical activity, grit, and depressive symptoms.

Grounded in resilience theory, this study proposes a conceptual model positioning grit as the mediating variable underpinning the pathway connecting physical activity and depressive symptoms (Fig 1).

## Methods

### Study design and participants

The present work is a cross-sectional study that seeks to examine grit as a potential mediator between physical activity and depressive symptoms in Chinese university students. This research plan was approved by the Ethics Review Committee of Anhui Normal University on May 28, 2025 (No: AHNU-ET 2025111)., consistent with the principles of the Declaration of Helsinki. All participants provided informed consent prior to survey initiation. Participants were assured of their rights, including privacy and voluntary participation. The corresponding author oversees the secure storage and management of all data—with encrypted storage systems and files—and access is limited solely to those with explicit authorization.

A convenience sampling strategy was employed to recruit full-time students from various Chinese universities between May 29 and June 20, 2025, aiming to obtain a comprehensive dataset for the analyses. The questionnaire survey process is as follows: First, the research team developed an electronic questionnaire using the Wenjuanxing platform (www.wjx.cn). All items were mandatory, requiring participants to respond to every question before submission, thereby ensuring no missing data. The questionnaire opened with an informed consent form on the first page. The second page contained detailed completion instructions, followed by standardized scales assessing physical activity, grit, and depressive symptoms. The survey concluded with a section requesting sociodemographic information (covering age, gender, academic year, major, and education level). Second, the questionnaire link was distributed to initial university contacts, who then helped disseminate it to student class or course groups to recruit voluntary participants. Finally, prior to the commencement of the survey, all participants were required to review the informed consent form. Checking the “agree to participate” option was considered equivalent to signing a written informed consent form and indicated their voluntary participation; participants were then directed to the formal survey section. Inclusion Criteria were: (a) formal enrollment as an adult student at a Chinese university; (b) good physical health over the past month; and (c) voluntary informed consent. Exclusion Criteria were: (a) current participation in continuing education or graduate (Master’s/PhD) programs; (b) a documented diagnosis from a hospital of severe mental illness (e.g., cognitive impairment, depression); and (c) being on a leave of absence exceeding six months. Initially, 3,215 respondents completed the online questionnaire employed in the present study. Following data quality screening, 75 responses were excluded due to aberrant response patterns (e.g., straight-lining) or implausible completion times (less than 4 minutes or exceeding 12 minutes), with thresholds based on pilot testing (n = 50; mean = 7.8 minutes, SD = 2.1 minutes) where the lower bound was set at half of the pilot mean and the upper bound at 1.5 times the pilot mean plus one standard deviation. No additional attention checks or data quality filters were used. Given the low exclusion rate (2.33%), the potential for selection bias was considered minimal. After exclusions, the study retained 3,140 valid responses for analysis, yielding a valid questionnaire response rate of 97.67%.

A priori sample size estimation was conducted using G*Power [67]. With a medium effect size (*f*^2^ = 0.15), significance level (*α*) of 0.05, statistical power of 0.95, and accounting for eight predictors, the minimum required sample size was determined to be at least 159 participants. With a total of 3,140 valid cases collected in the present study, the minimum sample size requirement was fully satisfied.

### Measures

#### Physical activity.

Physical activity levels were assessed using the Physical Activity Rank Scale-3 (PARS-3) [68], a valid instrument for measuring physical activity levels across different age groups, which was first translated and adapted by the Chinese scholar Liang [69] to suit Chinese samples. The scale comprises three continuous indicator items that quantify core dimensions of physical activity behavior over the most recent month: physical activity intensity, physical activity duration (the time sustained at a given intensity), and physical activity frequency (monthly occurrence of activity). Intensity and frequency items are rated on a 5-point scale, while duration is scored from 0 to 4. A composite physical activity index score (range: 0–100) is calculated by multiplying the raw scores of the three indicator items, and exercise volume classification follows Liang’s [69] original criteria (low: ≤ 19, moderate: 20–42, high: ≥ 43).

Given the PARS-3 yields a multiplicative composite index (derived from non-interchangeable indicator items of intensity, duration, and frequency), Cronbach’s α was calculated for the three raw indicator items to assess internal consistency of the scale’s constituent parts, rather than for the final composite index. In the present sample, the three indicator items demonstrated a Cronbach’s α of 0.634, which is consistent with the reliability range reported in prior Chinese studies using the adapted PARS-3 [70,71]. This range is considered statistically acceptable for brief scales (≤3 items) measuring multidimensional behavioral constructs like physical activity, where brevity may slightly constrain internal consistency [72]. The modest reliability coefficient may also be attributed to the inherent heterogeneity of physical activity behaviors (e.g., variations in activity types, individual differences in activity patterns) and the context-dependent nature of self-reported physical activity, which can introduce minor variability in item responses. Despite this, the PARS-3 was retained for its established validity, cultural adaptability, and consistent application in Chinese population studies [70,71], which supports its suitability for assessing physical activity in this study’s sample.

#### Grit.

University students’ grit was measured with the Short Grit Scale, a tool originally proposed by Duckworth and Quinn [73]. This two-dimensional instrument comprises 8 items (e.g., “I am diligent”). Responses were recorded on a 5-point Likert scale ranging from 1 (*very much unlike me*) to 5 (*very much like me*), with higher aggregate scores indicating stronger grit. The Chinese adaptation of the instrument has been employed on numerous occasions in prior research [27,74]. In the current sample, it showed a Cronbach’s alpha of 0.844.

#### Depressive symptoms.

To assess depressive symptoms among university students, we used the Patient Health Questionnaire-9 (PHQ-9). This tool, initially created by Kroenke et al. [75], is a widely validated brief instrument for screening and quantifying the severity of depressive symptoms, rather than for formal clinical diagnosis. Although originally designed for primary care settings, it has been extensively adopted in general population and college student surveys [76]. The nine-item scale includes items such as “Feeling down, depressed, or hopeless.” Participants rate symptom frequency over the preceding two weeks on a 4-point Likert scale ranging from 0 (“*Not at all*”) to 3 (“*Nearly every day*”). The total score had a minimum of 0 and a maximum of 27, with elevated scores signifying more pronounced depressive symptomatology. Consistent with established cutoff standards for Chinese young adults [77], a summed score of 11 or above was treated as indicating clinically relevant depressive symptoms based on screening criteria. When used to assess the Chinese college student population, the PHQ-9 has been proven to have good validity [77] and showed excellent internal consistency in the present study (α = 0.935).

#### Control variables.

Based on established empirical evidence, it is methodologically sound to statistically control for demographic characteristics when examining the relationships among physical activity, grit, and depressive symptoms.

Gender differences have been widely documented in these key variables. Males typically engage in significantly more physical activity than females [78,79], whereas females report notably higher levels of depressive symptoms, especially during the university period [80–82]. Findings regarding gender differences in grit have been mixed: female adolescents showed higher grit levels in a sample of 814 Israeli high school students [59], while male employees reported greater grit in a large sample of over 11,000 Korean workers [83].

Beyond gender, other demographic factors are also meaningfully related to the study variables. Age, academic year, and major were significantly associated with depressive symptoms among college students [84]. Academic year was negatively linked to physical activity, and educational level was positively associated with depressive symptoms in university students [70]. Similarly, Kwon et al. [85] confirmed significant differences in physical activity participation across educational levels.

Accordingly, adjusting for these demographic covariates in statistical analyses helps reduce confounding variance and supports a more accurate estimation of the focal relationships between physical activity, grit, and depressive symptoms.

### Statistical analysis

We conducted data analysis using SPSS 29.0 and Amos 29.0. A total of 3,140 participants with complete datasets were included in the final analyses. First, descriptive statistical analyses were performed on all demographic variables. Second, indicators of central tendency, variability, skewness, and kurtosis were calculated to examine the distributional properties of the key variables. Normality of the three main variables—physical activity, grit, and depressive symptoms—was evaluated using skewness and kurtosis values with a sample size of 3140. For physical activity, the skewness was 1.736 (SE = 0.044) and kurtosis was 2.689 (SE = 0.087), indicating a substantial positive skew and leptokurtic distribution. For grit, skewness was 0.993 (SE = 0.044) and kurtosis was 2.848 (SE = 0.087), suggesting a moderate positive skew. For depressive symptoms, skewness was 0.844 (SE = 0.044) and kurtosis was 1.260 (SE = 0.087), also reflecting a mild positive skew. Although all variables showed some degree of non-normality in univariate distributions, given the very large sample size, violations of normality were considered negligible for subsequent statistical analyses according to the central limit theorem. Therefore, robust nonparametric procedures were adopted in the primary analyses. Spearman’s rank correlation was applied to examine bivariate relationships in place of Pearson’s correlation, as it relies on rank-order information (rather than raw metric values) and thus is less sensitive to non-normal distributions [86]. Third, we evaluated the measurement model by performing confirmatory factor analysis (CFA) with Amos 29.0, assessed discriminant validity among the core constructs, and examined potential common method bias using Harman’s single-factor test. The measurement model was specified in accordance with the psychometric properties and factor structure of the original scales. The measurement model for each construct was explicitly specified based on its psychometric properties and scale structure:

Physical activity: Treated as a latent variable in the CFA, operationalized as the multiplicative composite score calculated from the three PARS-3 indicator items (intensity, duration, frequency). This specification was justified by the scale’s inherent design as a behavioral index with non-interchangeable dimensions, as a latent construct would not accurately reflect the multiplicative relationship of its constituent parts.

Grit: Treated as a latent variable with item-level indicators, comprising all 8 items of the Short Grit Scale [73]. All items were freely loaded onto the single grit latent factor, consistent with the scale’s unidimensional scoring framework in prior validation studies [27,74].

Depressive symptoms: Treated as a latent variable with item-level indicators, including all 9 items of PHQ-9 [75]. All items were freely loaded onto the single depressive symptoms latent factor, aligning with the scale’s validated unidimensional structure for measuring depressive symptom severity [76].

We compared the fit of three competing models: a single-factor model (all grit and PHQ-9 items, plus the physical activity observed index, loading on one common factor), a two-factor model (physical activity observed index + grit items as one factor; PHQ-9 items as a separate factor), and the proposed three-factor model (physical activity as an observed index, grit and depressive symptoms as distinct latent constructs, with all three constructs correlated). Model fit was evaluated using standard indices: χ², CFI, TLI, GFI, and RMSEA, with fit criteria from Ferreira-Valente et al. [87].

We tested our hypothesized mediation model—positing grit as a mediator in the relationship between physical activity and depressive symptoms—using Model 4 of the PROCESS macro (v4.1) in SPSS [88]. A bias-corrected bootstrapping procedure with 5,000 resamples was employed to assess the indirect effect’s significance, with a 95% confidence interval (CI) excluding zero indicating statistical significance [88].

## Results

### Descriptive statistics

The demographic composition of the final sample (N = 3,140) is presented in Table 1. Participants’ mean (M) age was 20.02 years (standard deviation, SD = 1.28), and ages ranged from 18 to 27. The sample consisted of 47.07% male and 52.93% female students. In terms of academic year, first-year, second-year, third-year, and fourth-year students represented 35.80%, 37.58%, 22.58%, and 4.04% of participants, respectively. The relatively low proportion of senior participants was attributed to the period of data collection (May to June), which coincides with the graduation season for Chinese college students—they were occupied with writing graduation theses and seeking employment during this time. In terms of academic background, liberal arts students represented 49.01% and science students 50.99%. Additionally, 24.24% of the participants were enrolled in 3-year post-secondary programs, and 75.76% in 4-year undergraduate programs.

**Table 1:** Sample demographics and descriptive statistics for demographic and core psychological variables (*n* = 3,140).

Variable	Category/Statistic	*n*(*%*)/Mean (SD)
Age	Range 18–27	20.02 (SD = 1.28)
Gender	1 = Male	1478 (47.07)
	2 = Female	1662 (52.93)
Academic year	1 = First-year student	1124 (35.80)
	2 = Second-year student	1180 (37.58)
	3 = Third-year student	709 (22.58)
	4 = Fourth-year student	127 (4.04)
Major	1 = Liberal arts	1539 (49.01)
	2 = Science	1601 (50.99)
Educational level^a^	1 = 3-year post-secondary programs	761 (24.24)
	2 = 4-year undergraduate programs	2379 (75.76)
Physical activity	Total sample	18.50 (SD = 21.37)
	low exercise volume	2,179 (69.39)
	moderate exercise volume	507 (16.15)
	high exercise volume	454 (14.46)
Depressive symptoms	Total sample	7.60 (SD = 5.25)
	PHQ-9 < 11	2536 (80.76)
	PHQ-9 ≥ 11	604 (19.24)
Grit	Total sample	25.21 (SD = 3.77)

On average, the participants’ physical activity level was recorded at 18.50 (SD = 21.37). Based on established physical activity criteria, 69.39% of the students were classified as having a low exercise volume, 16.15% as moderate, and 14.46% as high. The mean score for depressive symptoms on the PHQ-9 was 7.60 (SD = 5.25). Based on the established clinical threshold of ≥11, 80.76% of the students were categorized as having no clinically significant depressive symptoms, while 19.24% screened positive for clinically relevant depressive symptoms. Additionally, the mean grit score among the students was 25.21 (SD = 3.77).

### Preliminary analyses

To rule out potential common method bias across self-reported measures, we conducted the conventional single-factor exploratory factor analysis. A total of four latent components were extracted with eigenvalues above one, and the initial component contributed merely 33.66% to the overall explained variance, well below the 40% threshold [89]. Beyond the preceding tests, CFA was utilized under the structural equation modeling framework to probe existing common method bias [90]. A single-factor baseline model exhibited unsatisfactory fit, whereas the hypothesized three-factor measurement model achieved acceptable fit (Table 2). Based on these overall findings, severe common method bias was not detected within the present research sample, indicating that the identified associations were not driven by systematic measurement flaws in the survey tools [89]. Subsequently, multicollinearity among the independent variables was examined. The results demonstrated an absence of significant collinearity, with all variance inflation factor (VIF) values at 1.018, which indicates no cause for concern [91].

**Table 2:** Model fit results for three candidate measurement structures.

Model	*χ* ^*2*^	*χ* ^*2*^ */df*	*CFI*	*TLI*	*GFI*	*RMSEA*
Model 1	15914.939	93.617	0.566	0.515	0.595	0.172
Model 2	10072.682	59.602	0.727	0.693	0.699	0.137
Model 3	264.009	2.614	0.996	0.992	0.992	0.023

Model 1: All items from PARS-3, Short Grit Scale, and PHQ-9 loaded on a single factor. Model 2: PARS-3 and Short Grit Scale items were combined into one factor, with PHQ-9 as a separate factor. Model 3: PARS-3, Short Grit Scale, and PHQ-9 were modeled as three correlated yet distinct latent variables.

Using Amos 29.0, we performed three CFAs to compare theoretically derived measurement models, selecting the three-factor model as the best-fitting solution (Table 2). Its fit indices met recommended thresholds [87], supporting the discriminant validity and structural integrity of the three-factor construct.

### Correlation analysis

Descriptive statistics, internal consistency estimates, and inter-variable correlations for all measures are summarized in Table 3. For the latent variables (physical activity, grit and depressive symptoms), psychometric properties were examined via composite reliability (CR) and average variance extracted (AVE): all constructs exhibited robust convergent validity, with CR indices surpassing 0.70 and AVE estimates exceeding the 0.50 benchmark [87]. Discriminant validity was confirmed using the Fornell–Larcker criterion [92]. For all latent constructs, the square root of each construct’s AVE (diagonal values in Table 3) was greater than its correlation coefficients with all other latent constructs.

**Table 3:** Spearman rank correlations for the study sample (*n* = 3,140).

Variables	M ± SD	α	AVE	CR	1	2	3	4	5	6	7	8
1 Age	20.02 ± 1.28				1							
2 Gender	1.53 ± 0.50				−0.002	1						
3 Academic year	1.95 ± 0.86				0.762***	0.062***	1					
4 Educational level	1.76 ± 0.43				0.193***	0.202***	0.206***	1				
5 Major	1.51 ± 0.50				−0.062***	−0.358***	−0.024	−0.097***	1			
6 Physical activity	18.50 ± 21.37	0.634	0.575	0.800	−0.054**	−0.273***	−0.099***	0.029	0.018	**0.758**		
7 Grit	25.21 ± 3.77	0.844	0.517	0.895	0.032	0.040*	0.018	0.015	−0.025	0.165***	**0.719**	
8 Depressive symptoms	7.60 ± 5.25	0.935	0.641	0.941	−0.011	−0.025	0.002	0.042*	0.007	−0.089***	−0.361***	**0.801**

Bolded diagonal values are square roots of AVE for discriminant validity assessment. Cronbach’s α, AVE, and CR reflect internal consistency and convergent validity. *p < 0.05, **p < 0.01, and ***p < 0.001.

Spearman’s correlation analyses (n = 3,140) were conducted for the three core study variables. As shown in Table 3, physical activity showed a weak positive association with grit (*ρ* = 0.165, *p* < 0.001), while it correlated mildly and inversely with depressive symptoms. (*ρ* = −0.089, *p* < 0.001). Grit exhibited a moderate negative association with depressive symptoms (*ρ* = −0.361, *p* < 0.001). Although the bivariate correlation between physical activity and depressive symptoms was very small (*ρ* = −0.089), this association carries meaningful implications at the population level. In clinically interpretable units, a one-standard-deviation increase in physical activity corresponded to a 0.47-point decrease in PHQ-9 depressive symptom scores (calculated as *ρ* × SD of PHQ-9).

### Grit as a mediator

**Fig 2:**
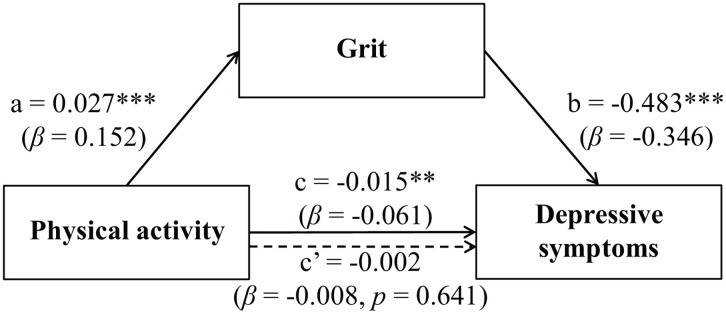
Path coefficients for the mediated relationship of physical activity with depressive symptoms through grit. Path values are unstandardized regression coefficients, with standardized coefficients (*β*) reported in parentheses. The total association between physical activity and depressive symptoms is denoted as path c, the direct association (adjusted for grit) as path c’, and the two components of the indirect association via grit as paths a and **b.** The indirect association (*a × b*) was evaluated using 5,000 bias-corrected bootstrap resamples to account for non-normality. All paths control for age, gender, academic year, educational level, and major. **p < 0.01; ***p < 0.001.

Controlling for age, gender, academic year, educational level, and major, mediation analysis demonstrated that physical activity was indirectly and significantly linked to lower depressive symptoms via grit. As shown in Table 4 and Fig 2, path analysis results indicated a positive association between physical activity and grit (**path a**): *a* = 0.027, *p* < 0.001, 95% CI [0.020, 0.033], and grit was inversely related to depressive symptoms (**path b**), *b* = −0.483, *p* < 0.001, 95% CI [−0.529, −0.437]. Bias-corrected bootstrap resampling with 5,000 iterations confirmed a significant indirect association (*a × b*) between physical activity and depressive symptoms through grit, *ab* = −0.013, 95% CI [−0.016, −0.010]. Physical activity demonstrated a statistically significant, inverse total association with depressive symptoms (**path c**), *c* = −0.015, *p* < 0.01, 95% CI [−0.024, −0.006], whereas the direct association was not significant after accounting for grit (**path c’**), *c’* = −0.002, *p* > 0.05, 95% CI [−0.011, 0.007]. These findings confirm grit as a mediating variable linking physical activity to depressive symptoms among Chinese university students.

**Table 4:** Parameter estimates for the mediation model of physical activity, grit, and depressive symptoms.

Predictors	Grit			Depressive symptoms		
	*β*	*SE*	*95%CI*	*β*	*SE*	*95%CI*
Age	0.135	0.078	[-0.018, 0.287]	−0.157	0.103	[-0.358, 0.044]
Gender	0.542***	0.153	[0.242, 0.842]	−0.588**	0.202	[-0.985, -0.192]
Academic year	−0.129	0.116	[-0.356, 0.098]	0.177	0.153	[-0.123, 0.477]
Educational level	−0.177	0.163	[-0.497, 0.143]	0.642**	0.215	[0.220, 1.064]
Major	0.045	0.144	[-0.238, 0.328]	−0.150	0.190	[-0.523, 0.223]
Physical activity	0.027***	0.003	[0.020, 0.033]	−0.002	0.004	[-0.011, 0.007]
Grit				−0.483***	0.024	[-0.529, -0.437]
R^2^	0.023			0.127		
F	12.192***			64.797***		

**p < 0.01; ***p < 0.001. SE, Standard Error.

Using a bootstrap approach with 5,000 resamples, we examined how well our associational findings held under non‑normal data distributions. The generated 95% percentile confidence intervals (CIs) corroborated the persistence of each statistically significant association. Specifically, three associations maintained their statistical significance, as evidenced by 95% CIs that did not contain zero: physical activity with grit (*ρ* = 0.165, 95% CI [0.129, 0.199]), physical activity with depressive symptoms (*ρ* = −0.089, 95% CI [−0.125, −0.053]), and grit with depressive symptoms (*ρ* = −0.361, 95% CI [−0.392, −0.329]).

For the assessment of indirect associational patterns, bias-corrected and accelerated (BCa) bootstrap intervals were adopted to accommodate non-normal data distributions. The indirect link between physical activity and depressive symptoms via grit maintained statistical significance, with a minor effect magnitude: *ab* = −0.013, 95% BCa CI [−0.016, −0.010]. In fully standardized coefficients, this indirect association corresponded to *β*_indirect_ = −0.053, 95% BCa CI [−0.067, −0.039]. Bootstrap confidence intervals for the primary pathways (physical activity to grit, and grit to depressive symptoms) were concordant with the baseline results outlined in Table 4. Overall, these analyses verify that both the bivariate correlational trends and the indirect associational framework remain stable across violations of normality assumptions.

## Discussion

The present cross-sectional study examined the relationship between physical activity and depressive symptoms among 3,140 Chinese university students, with grit as a potential mediator. The results fully supported both proposed hypotheses: physical activity was negatively associated with depressive symptoms, and grit served as a complete mediator in this relationship. These findings align with and extend existing literature, while shedding light on the unique psychological mechanisms underlying the mental health benefits of physical activity among young adults in the Chinese cultural context.

### Interpretation of findings

Hypothesis 1, which posits a negative association between physical activity and depressive symptoms, is consistent with a large body of empirical evidence [53–55]. To explain these observed associations, researchers have explored the underlying mechanisms from multiple perspectives. From a biological perspective, physical activity stimulates the release of neurotransmitters such as dopamine and serotonin, thereby enhancing mood and reducing susceptibility to conditions like anxiety and depression [93,94]. From a psychological perspective, physical activity can effectively regulate attention [95], help individuals break free from the distress of negative emotions [96], and enhance self-efficacy and self-confidence [30,97]. On the social front, participation in physical activities also offers college students opportunities for social interaction and helps broaden their social networks [98], thereby strengthening perceived social support [99] and contributing to enhanced psychological resilience and greater well-being [100,101]. From a behavioral perspective, regular physical activity promotes sleep quality and reduces sedentary behaviors [102,103]—both of which are established risk factors for depression among young adults [104].

Hypothesis 2 was also supported, as grit was found to mediate the relationship between physical activity and depressive symptoms, even though the mediated effect size was relatively small. This finding contributes novel insights to the literature by identifying grit as a key psychological factor associated with both physical activity and mental health outcomes. Consistent with our results, Duckworth et al. [105] argued that grit—defined as passion and perseverance for long-term goals—enables individuals to cope with adversity and maintain emotional stability, thereby reducing vulnerability to depression. A study by Okafor et al. [106] further demonstrated that higher levels of perseverance can reduce the severity of stress, anxiety and depression. Several associations may explain the link between grit, physical activity, and depressive symptoms. First, physical activity is inherently associated with higher levels of grit, as engagement in physical activity involves repeated experiences of goal-setting, effort investment, and overcoming challenges [28,29]. Over time, these experiences are associated with enhanced capacity to persist in the face of difficulties, a core component of grit. Second, the association between grit and physical activity is further reflected in the tendency for individuals with high levels of grit to engage in consistent physical activity. Specifically, individuals with high levels of grit are more likely to sustain long-term physical activity engagement, as they are less prone to abandoning healthy behaviors when faced with common barriers (e.g., weather conditions, sports-related injuries, heavy academic pressure, insufficient time and energy, or low motivation for exercise [60]), which in turn is associated with the maintenance of mental health benefits from such activity [107]. Third, grit is directly associated with lower levels of depressive symptoms, partly through its association with a growth mindset and perceived control [108,109]. Individuals with high grit tend to view challenges as opportunities for growth rather than insurmountable obstacles, which is associated with greater resilience and reduced helplessness [41]. This mental framework aligns with resilience theory, which posits that optimism (e.g., perceived autonomy and competence) is critical for mental health—for example, by enhancing well-being, buffering against the negative effects of stress, and reducing the risk of post-traumatic stress disorder [41].

### Theoretical Contributions

The present study makes several notable theoretical contributions to the existing literature by examining the mediating role of grit in the relationship between physical activity and depressive symptoms among Chinese university students, grounded in the resilience theory. These findings not only validate the applicability of resilience theory in explaining mental health outcomes among young adults in a Chinese cultural context but also extend the theoretical understanding of the mechanisms linking health behaviors to emotional well-being.

First, this study enriches the resilience theory by identifying grit as a critical psychological resource that translates physical activity into reduced depressive symptoms. Resilience theory posits that individuals with enhanced psychological resilience are better equipped to cope with adversity and maintain emotional stability [41]. While previous research has highlighted factors such as resilience, self-efficacy, adaptive capacities, and social support as key protective resources [40], our findings complement this framework by demonstrating that grit serves as a distinct and actionable mechanism. Specifically, the mediation effect of grit indicates that physical activity is associated with resilience through its relationship with grit, which in turn is linked to lower depressive symptoms. This aligns with Duckworth et al. [105], who argued that grit enhances an individual’s capacity to persist in the face of challenges, a core component of psychological resilience. Extending this line of research, our study provides empirical evidence linking grit to both physical activity engagement and reduced depressive symptoms, highlighting a behavioral and psychological pathway consistent with resilience theory.

Second, the study extends the literature on the physical activity–mental health nexus by uncovering a unique mediating pathway that is particularly relevant to Chinese university students. Prior meta-analyses have consistently established a negative association between physical activity and depressive symptoms [110,111], but the underlying psychological mechanisms remain underexplored, especially in non-Western contexts. Most existing studies have focused on biological pathways (e.g., inflammation, oxidative stress, or neuroendocrine system [112]) or general psychological factors (e.g., psychological inflexibility [113]), with limited attention to character strengths like grit. Our finding of mediation suggests that the association between physical activity and mental health is explained, at least in part, by grit, indicating that these variables are interlinked in a coherent pattern consistent with resilience theory.

Third, this study contributes to the discourse on resilience theory by examining the role of grit as a malleable psychological resource associated with physical activity. Contemporary resilience frameworks emphasize that resilience emerges within specific contexts and evolves across life stages—it is a dynamic process shaped by modifiable reserve capacities and contextual factors, rather than a fixed trait [41]. While prior research has established the conceptual link between grit and resilience [114,115], and some evidence has connected physical activity to both constructs [37,116], few studies have explicitly positioned grit as a mediator in the relationship between physical activity and reduced depressive symptoms—an important extension of resilience theory. Notably, our findings align with and extend previous research [41] demonstrating that non-cognitive skills like grit are not immutable; rather, they may develop in conjunction with health-related behaviors such as physical activity [37]. For university students, who frequently confront academic stress, career uncertainty, and interpersonal pressure [46–48], identifying modifiable factors associated with resilience is crucial. Dunston et al. [28] found a positive association between vigorous physical activity and both resilience and the perseverance-of-effort dimension of grit among university students. Extending this line of research, our study illustrates that grit is linked to both physical activity and reduced depressive symptoms, supporting the core tenet of resilience theory regarding how modifiable resources (e.g., grit, in conjunction with physical activity) may interact with contextual stressors in relation to mental health trajectories.

Fourth, the study addresses a gap in the literature by examining the mediating role of grit in a large, culturally homogeneous sample. Most previous studies on grit and mental health have been conducted in Western contexts [58,105], with limited empirical evidence from East Asia. Cultural values, such as collectivism and emphasis on perseverance, may shape the expression and correlates of grit [34]. Our findings indicate that grit operates similarly as a mediator in the Chinese cultural context, but with a stronger effect size, suggesting that cultural values may be associated with an amplified role of grit in relation to mental health. This aligns with Jung et al. [83], who found that grit moderated the relationship between occupational stress and mental health outcomes among Korean workers. By integrating cultural context into the theoretical model, our study contributes to a more inclusive understanding of grit’s associations with mental health and underscores the need for cross-cultural comparative research.

In summary, the present study makes significant theoretical contributions by validating grit as a mediator in the physical activity–depressive symptoms relationship, extending resilience theory, providing cross-cultural insights, and integrating positive psychology with public health. These findings not only contribute to our understanding of the psychological processes associated with the relationship between physical activity and mental health but also provide a basis for further research into interventions that combine physical activity promotion and grit training.

### Implications for practice

While the bivariate associations identified in this study appear modest at the individual level, they carry meaningful population-level implications for public health and student wellness programs: each standard-deviation increase in physical activity was associated with an approximately 0.47-point reduction in depressive symptoms. Although such effect magnitudes seem small on an individual basis, they gain clinical relevance when extended to large student populations, suggesting that promoting physical activity may represent a gentle yet meaningful population-level approach to supporting mental health and alleviating the overall burden of depressive symptoms among university students. Notably, statistical significance does not imply substantial practical effects; the large sample in this study (N = 3,140) allowed for the detection of small but robust associations, including a small but statistically reliable indirect association between physical activity, grit, and depressive symptoms (*β* = −0.013), which aligns with population-level mental health benefits rather than pronounced clinical changes at the individual level. Nevertheless, these modest associations remain meaningful for preventive interventions among university students, as gradual improvements in mental health indicators can reduce the aggregate burden of depressive symptoms at scale.

The confirmed negative correlation between physical activity and depressive symptoms, with grit as a mediator, holds crucial practical implications for Chinese university students—particularly given the low rate of professional mental health help-seeking among this group. Research consistently indicates that university students with depressive symptoms worldwide, including East Asian populations, often avoid formal psychological support due to stigma, perceived inaccessibility, or cultural norms emphasizing self-reliance [117]. As physical activity is a low-cost, side-effect-free, and accessible intervention to alleviate stress and promote mental health among university students [118,119], universities should promote it as a proactive mental health strategy tailored to students’ needs. Given that structured, sustained physical activity fosters grit and passion [31,105], universities could offer accessible, flexible programs (e.g., lunchtime group workouts, virtual fitness challenges) to accommodate busy academic schedules. Mental health counselors should integrate physical activity recommendations into informal support interactions, framing physical activity as a evidence-based tool to build grit and reduce distress. Policymakers and student affairs teams should also address systemic barriers by expanding access to fitness facilities and offering free, beginner-friendly classes (e.g., low-intensity aerobic exercise, mindfulness-based movement). Such an approach empowers students to take an active role in their mental well-being through a culturally acceptable and scalable strategy that complements the utilization of limited professional support. Furthermore, when promoting physical activity among university students, it is crucial to account for gender-based differences in exercise participation. Tailored, personalized strategies are recommended, moving beyond one-size-fits-all policy recommendations [120]. Furthermore, mental health education curricula could integrate grit-training modules designed to stimulate university students’ intrinsic motivation and foster self-efficacy. These core psychological assets can act in concert with the benefits derived from physical activity, collectively contributing to prevent or alleviate depressive symptoms in the university student population [31].

### Limitations and future research

Several methodological constraints should be considered when interpreting these results. Firstly, the cross-sectional nature of the data limits conclusions about directional or causal pathways among physical activity, grit, and depressive symptoms. Employing longitudinal or experimental approaches in subsequent studies would help clarify the temporal sequence and potential causal mechanisms. Secondly, although statistical tests were conducted to assess common method bias, all variables were collected via self-reported questionnaires at a single time point, which still carries a potential risk of common method variance. Such bias may artificially inflate or attenuate the observed relationships. Future studies are encouraged to collect data from multiple sources, use time-lagged designs, or include marker variables to further control for common method bias. Thirdly, the study used a non-probability convenience sample recruited through social networks, which may restrict the generalizability of the findings [121]. Moreover, since the sample comprised only Chinese university students, the cultural specificity of the results should be noted. Future studies employing stratified random sampling and cross-cultural designs would improve representativeness and enable broader cross-contextual validation. Fourthly, the internal consistency coefficient of the Physical Activity Rank Scale-3 used in this study was relatively low (Cronbach’s α = 0.634), slightly below the conventional threshold of 0.70. This may introduce random measurement error, which could attenuate the strength of the observed associations between physical activity and other variables and reduce the precision of the study results. For future research, it is recommended to adopt more comprehensive and well-validated physical activity scales with established high internal consistency, or revise and optimize the existing scale items to improve its reliability, thereby enhancing the accuracy and robustness of the measurement results. Notably, the current mediation model showed limited explanatory power, accounting for only 2.3% of variance in grit (R² = 0.023) and 12.7% in depressive symptoms (R² = 0.127), suggesting the model omits key factors contributing to these outcomes. Future research should incorporate additional predictors to improve explanatory power. Finally, it is important to note that the association between physical activity, perseverance and depressive symptoms observed in this study is relatively small in magnitude. Although it is statistically significant due to the large sample size, these effects reflect moderate changes at the individual level and should be interpreted as preventive benefits for the entire population, rather than strong clinical outcomes. Future research may explore larger effect sizes and clinical results to enhance their practical application value.

## Conclusions

This study provides preliminary evidence that grit, as a psychological resource, mediates the association between physical activity and depressive symptoms among Chinese university students. These results indicate that the mental health benefits linked to physical activity are not merely reflective of physiological processes, but are also closely tied to the cultivation of perseverance and passion for long-term goals. The findings support a more comprehensive approach to student well‑being that integrates physical health promotion with the development of key character strengths, thereby supporting psychological resilience in relation to depression.
